# Supplementary material for: European Markets for Cultured Meat: A Comparison of Germany and France
Source: Foods. 2020 Aug 21;9(9):1152. doi: 10.3390/foods9091152 (PMC7555944; doi:10.3390/foods9091152)
Supplement: Supplementary file 1 [file foods-09-01152-s001.pdf]

CLEAN MEAT SURVEY

ASK ALL. **QUOTA**

S1. BIRTHDAY [Year/Month]

What is your date of birth?

YEAR/MONTH DROPDOWN

ASK ALL. SINGLE.

S2. GENDER **QUOTA**

Are you?

1. Male
2. Female

ASK ALL

S3. REGION **QUOTA**

Where do you live?

INTRODUCTION

Thank you for taking part in this survey.

The survey will take around 15 minutes. Your opinion is very important to us because your answers will help us understand how people in different countries think about various topics. Therefore, we would like to ask you to answer the questions without interruption, if possible.

Your participation is voluntary. Your answers will be treated completely anonymously and confidentially and will only be analyzed for statistical purposes.

Please click on "next" to start the survey.

MAIN QUESTIONNAIRE

ASK ALL. SINGLE

Q1. SENSITIVITY ENVIRONMENT

Please indicate how much you agree with the following statement: I feel concerned about environmental issues.

1 Completely agree
2 Agree
3 Neither agree nor disagree
4 Disagree
5 Completely disagree
97 Don't know/ Prefer not to say

ASK ALL. SINGLE

Q2. SENSITIVITY ANIMAL WELFARE

Please indicate how much you agree with the following statement: I feel concerned about animal welfare.

1 Completely agree
2 Agree
3 Neither agree nor disagree

4 Disagree
5 Completely disagree
97 Don’t know/ Prefer not to say

ASK ALL. SINGLE
Q3. SENSITIVITY WORLD HUNGER
Please indicate how much you agree with the following statement: I feel concerned about food security / world hunger.
1 Completely agree
2 Agree
3 Neither agree nor disagree
4 Disagree
5 Completely disagree
97 Don’t know/ Prefer not to say

ASK ALL. SINGLE
Q4. SENSITIVITY FOOD SAFETY HEALTH
Please indicate how much you agree with the following statement: I feel concerned about the safety of food products for my own health (ex: contamination of food by pathogens).
1 Completely agree
2 Agree
3 Neither agree nor disagree
4 Disagree
5 Completely disagree
97 Don’t know/ Prefer not to say

Show all
Q5 Infotext
Please have a look at the following definitions of diet types.
- Omnivore (Eats meat, fish/seafood, eggs, dairy etc)
- Flexitarian (Limits the consumption of meat and/or fish/seafood)
- Pescatarian (Eats fish/seafood, eggs and dairy but no meat)
- Vegetarian (Does not eat meat or fish/seafood but eggs and dairy)
- Vegan (Does not eat meat, fish/seafood, dairy and eggs)

ASK ALL. SINGLE
Q6. DIETARY IDENTITY
After having read the definitions of diet types, how would you describe yourself?
1 Omnivore
2 Flexitarian
3 Pescatarian
4 Vegetarian
5 Vegan
97 Don’t know/ Prefer not to say

ASK IF Q6=1,2 (EATS MEAT). SINGLE
Q7. FREQUENCY MEAT CONSUMPTION
How often do you consume meat (any type of meat including: beef, pork, chicken, turkey; in any form: steaks to sausages)? Think about all the meals and snacks you eat. Also think about everything you have at home or outside the home.
1 2 or more times per day
2 Once per day
3 5-6 times per week

4 2-4 times per week
5 Once per week
6 2-3 times per month
7 Once per month
8 Less than once per month

ASK IF Q6=1,2 (EATS MEAT). SINGLE
Q8. INTENTION TO REDUCE MEAT
Do you intend to reduce your consumption of meat?
1 Definitely yes
2 Probably yes
3 Unsure
4 Probably no
5 Definitely no
97 Don't know/ Prefer not to say

ASK IF Q6=,3,4,5 OR Q8=1,2 (IS FLEXITARIAN, INTENDS TO REDUCE OR DOES NOT EAT MEAT). MULTI
Q9. REASONS FOR REDUCING MEAT
What are the reasons for not consuming meat, reducing or for having the intention to reduce your meat consumption? Please select all the answers that apply.
Meat…
RANDOMIZE
1 is expensive
2 is not animal welfare friendly
3 is not healthy
4 has a negative impact on the environment
5 is unsafe (antibiotics, contaminants in meat)
6 FIX I don't like meat
7 FIX Other

Q10 SHOW ALL
INFO TEXT
A few years ago, the first piece of meat was grown directly from cow cells, without slaughtering or even harming the animal. By sampling the cells which are responsible to renew muscle tissues and growing them under controlled conditions – it was proved that the same meat that grows inside the animal, can grow also outside of it. Food companies and universities around the world are currently working on perfecting the production method of this kind of meat, which is expected to reach your plate within the next 3 to 4 years.

ASK ALL. OPEN
Q11. NOMENCLATURE
Which name do you think would describe this meat?
… [open]

ASK ALL. SINGLE
Q12. FAMILIARITY
Had you heard of this kind of meat before?
1 No
2 Yes, but I didn't know what exactly it was
3 Yes, and I knew what it is

ASK ALL. SINGLE
Q13. FAVOURABILITY OF PRODUCTION

Are you in favor of the production of this cell-based type of meat, often called "cultured meat"?
1 Definitely in favor
2 Probably in favor
3 I am neither in favor, nor against
4 Probably against
5 Definitely against
97 Don’t know/ Prefer not to say

ASK ALL. SINGLE
Q14. WILLINGNESS TO TRY (AFTER INFO)
Would you be willing to taste/ try cultured meat?
1 Definitely yes
2 Probably yes
3 Probably no
4 Definitely no
97 Don’t know/ Prefer not to say

ASK IF Q6=2-4 (DOES NOT EAT MEAT). SINGLE
Q15. WILLINGNESS INCORPORATE (VEG)
Would you incorporate this meat into your diet?
1 Definitely yes
2 Probably yes
3 Probably no
4 Definitely no
97 Don’t know/ Prefer not to say

Show all
Q16 Infotext
For the following questions, please assume that cultured meat has the same taste as conventional meat.

ASK IF Q6=1,2 (EATS MEAT). SINGLE
Q17. WILLINGNESS TO PAY MORE (MEAT-EATERS)
Would you be willing to pay a higher price for cultured meat than for conventional meat?
1 Yes
2 No
97 Don’t know/ Prefer not to say

ASK IF Q6=2-4 (DOES NOT EAT MEAT). SINGLE
Q18. WILLINGNESS TO PAY MORE (NON MEAT-EATERS)
Would you be willing to pay a higher price for cultured meat than for f.ex. plant-based meat alternatives?
1 Yes
2 No
97 Don’t know/ Prefer not to say

ASK IF Q17=1 or Q18=1 (MEAT EATERS OR VEG*ANS WHO WOULD PAY MORE)
Q19. AMOUNT TO PAY MORE
What is the maximum price you would be willing to pay for a 100 grams piece of cultured meat, if for comparison, the cost of conventional meat was 1€ per 100 grams?
1 1,10 €

2 1,20 €
3 1,50 €
4 2,50 €
5 5,00 € or more
97 Don’t know/ Prefer not to say

Show all
Q20 Infotext
For the following questions, please assume that cultured meat not only tastes like conventional meat but also that the price is the same.

ASK ALL
Q21. WILLINGNESS TO BUY
Would you be willing to buy cultured meat when available in stores?
1 Definitely yes
2 Probably yes
3 Unsure
4 Probably no
5 Definitely no
97 Don’t know/ Prefer not to say

ASK IF Q6=1,2 (EATS MEAT). SINGLE
Q22. WILLINGNESS TO REPLACE (GENERAL)
Would you be willing to replace a part of your conventional meat with cultured in your daily diet?
1 Definitely yes
2 Probably yes
3 Unsure
4 Probably no
5 Definitely no
97 Don’t know/ Prefer not to say

ASK IF Q22=1,2 (WOULD REPLACE)
Q23. WILLINGNESS TO REPLACE (GMO)
Would you be willing to replace a part of your conventional meat with cultured meat that was GENETICALLY MODIFIED/THAT CONTAINS GMOs, in your daily diet?
1 Definitely yes
2 Probably yes
3 Unsure
4 Probably no
5 Definitely no
97 Don’t know/ Prefer not to say

ASK IF Q22=1,2 (WOULD REPLACE)
Q24. WILLINGNESS TO REPLACE (NON-GMO)
Would you be willing to replace a part of your conventional meat with cultured meat that was NOT GENETICALLY MODIFIED /THAT DOES NOT CONTAIN GMOs, in your daily diet?
1 Definitely yes
2 Probably yes
3 Unsure
4 Probably no

5 Definitely no
97 Don't know/ Prefer not to say

SHOW IF Q6=1,2 (EATS MEAT).
Q25. Infotext
Please continue to assume that the taste and price for cultured meat are the same as for clean meat.

ASK IF Q6=1,2 (EATS MEAT). SINGLE
Q26. PRESSURE POINT ENVIRONMENT
Would knowing that cultured meat reduces the environmental impact of agriculture make you replace a part of your conventional meat with cultured meat?

1 It would definitely motivate me
2 It would probably motivate me
3 It would probably not motivate me
4 It would definitely not motivate me
97 Don't know/ Prefer not to say

ASK IF Q6=1,2 (EATS MEAT). SINGLE
Q27. PRESSURE POINT ANIMAL WELFARE
Would knowing that cultured meat doesn't involve the killing of an animal motivate you to replace a part of your conventional meat with cultured meat?

1 It would definitely motivate me
2 It would probably motivate me
3 It would probably not motivate me
4 It would definitely not motivate me
97 Don't know/ Prefer not to say

ASK IF Q6=1,2 (EATS MEAT). SINGLE
Q28. PRESSURE POINT FOOD SAFETY
Would knowing that cultured meat reduces the risk of contamination by pathogens motivate you to replace a part of your conventional meat with cultured meat?
1 It would definitely motivate me
2 It would probably motivate me
3 It would probably not motivate me
4 It would definitely not motivate me
97 Don't know/ Prefer not to say

ASK IF Q6=1,2 (EATS MEAT). SINGLE
Q29. PRESSURE POINT ANTIBIOTICS
Would knowing that no antibiotics are used in the production of cultured meat motivate you to replace a part of your conventional meat with cultured meat?
1 It would definitely motivate me
2 It would probably motivate me
3 It would probably not motivate me
4 It would definitely not motivate me
97 Don't know/ Prefer not to say

ASK ALL. SINGLE
Q30. PREFERENCE OVER PLANT-BASED MEAT

Would you rather eat cultured meat than plant-based (peas, seitan, soy, lentils) alternatives to meat?
I would…
1 strongly prefer cultured meat over plant-based meat
2 somewhat prefer cultured meat over plant-based meat
3 somewhat prefer plant-based meat over cultured meat
4 strongly prefer plant-based meat over cultured meat
5 eat both
6 not eat either
97 Don't know/ Prefer not to say

ASK ALL. SINGLE
Q31. PREFERENCE OVER INSECT-BASED MEAT
Would you rather eat cultured meat than insect-based alternatives to meat?
I would…
1 strongly prefer cultured meat over insect -based meat
2 somewhat prefer cultured meat over insect -based meat
3 somewhat prefer insect -based meat over cultured meat
4 strongly prefer insect -based meat over cultured meat
5 eat both
6 not eat either
97 Don't know/ Prefer not to say

ASK ALL. OPEN
Q32. THOUGHTS ON CULTURED MEAT
What kinds of thoughts do you have about cultured meat? (This includes unfinished thoughts, such as doubts, worries, hopes, mixed feelings...).
… [open]

ASK ALL. SINGLE
Q33. NAME PREFERENCE
Which name out of these options do you prefer for this meat that we mentioned earlier?
RANDOMIZE
GERMAN
1
2
3
4
5
6
7
8
9
10
11
97 Don't know/ Prefer not to say

FRENCH
1
2

3
4
5
6
7
8
9
10
11
97 Don’t know/ Prefer not to say

DEMOGRAPHIC QUESTIONS

SHOW ALL.
Please answer a few questions about yourself to finish the questionnaire. (*country specific GDPR text included here)*

ASK ALL.
D1. EDUCATION
What is the highest level of education you achieved?

GER
1 Keine Schulausbildung
2 Mittel-/ Real-/ Höhere-/ Fach-/ Handelsschule, ohne Abschluss
3 Mittel-/ Real-/ Höhere-/ Fach-/ Handelsschule
4 Höhere Schule/Gymnasium, ohne Abschluss
5 Abitur/ Hochschulreife
6 Hochschulausbildung, ohne Abschluss
7 Universitäts-/ Fachhochschulabschluss
97 Keine Angabe

FR
1 École Élémentaire
2 Collège (BREVET)
3 CAP, BEP
4 Baccalauréat
5 Bac + 2 (BTS, DEUG, DUT)
6 Bac +3/+4 (Licence, Maîtrise, Master 1)
7 Bac +5 et plus (Grandes Écoles, DESS, DEA, Doctorat, Master 2)
97 Je préfère ne pas répondre

ASK ALL.
D2. EMPLOYMENT STATUS
Which of the following best describes your current situation?
SINGLE.
1 Employed - full time (32 or more hours weekly)
2 Employed - part time (15 to less than 32 hours weekly)
3 Employed - less than 15 hours
4 Unemployed

5 Helping family members (unpaid)
6 In vocational training
7 Student, in school
8 Retired
9 Doing housework, looking after children or other persons
10 In community or military service
11 Permanently sick or disabled
12 Other
97 Prefer not to say

ASK ALL.

D3. WORK IN ANIMAL AGRICULTURE

Are you working in the animal agriculture or meat production industries (livestock farmer, butcher, slaughterhouse worker, breeder, cattle feed supplier, meat processor, BBQ restaurant…)?
1 Yes
2 No
97 Don't know/ Prefer not to answer

ASK ALL.

D4. POLITICAL VIEWS

In politics, people sometimes speak about "left" and "right." Where would you place yourself on a scale from 1 to 10, with 1 meaning "very left" and 10 meaning "very right?"
1 Very left
2
3
4
5
6
7
8
9
10 Very right
97 Don't know/ Prefer not to say

ASK ALL.

D5. INCOME

What is your monthly income?
1 Less than 1 000 euros
2 Between 1 000 euros and less than2 000 euros
3 Between 2 000 euros and less than 3 000 euros
4 Between 3 000 euros and less than 4 000 euros
5 More than 4 000 euros
97 Don't know/ Prefer not to answer

SHOW ALL.

Thank you for your participation!
